# School-based HPV Vaccination: The Challenges in a Brazilian Initiative

**DOI:** 10.1055/s-0041-1740279

**Published:** 2021-12-21

**Authors:** Julio Cesar Teixeira, Mariana Silva Castro Vianna, Diama Bhadra Vale, Daniella Moretti Arbore, Thais Helena Wilmers Perini, Tulio Jose Tomass Couto, Jose Pedroso Neto, Luiz Carlos Zeferino

**Affiliations:** 1 Department of Obstetrics and Gynecology, Faculty of Medical Sciences, Universidade Estadual de Campinas, Campinas, SP, Brazil; 2 Epidemiological Surveillance and Women Health Secretariat, Administration of the City of Indaiatuba – Mayor's Office, Indaiatuba, SP, Brazil

**Keywords:** human papillomavirus, HPV vaccine, adolescents, schools, vaccination, papilomavírus humano, vacina contra HPV, adolescentes, escolas, vacinação

## Abstract

**Objective** The present study assesses the implementation and the impact after 2 years of a school-based *human papillomavirus* (HPV) vaccination program in a Brazilian city.

**Methods** A prospective study assessing the implementation of the program, offering quadrivalent HPV vaccine in two annual doses to girls and boys aged from 9 to 10 years old. The program was started in the city of Indaiatuba, state of São Paulo, Brazil, in 2018, and had authorization from the National Immunization Program. The number of HPV vaccine first doses applied and the coverage in 2018 was calculated and compared to the year 2017. There were described events that have influenced the results.

**Results** The program invited 4,878 children through schools (87.1% of the target population), and 7.5% refused vaccination. Several concurrent events required or competed for health professionals of the vaccination teams. The coverage of the first dose (between 9 and 10 years old) was 16.1% in 2017 and increased to 50.5% in 2018 ( *p* < 0.0001). The first dose in all ages increased 78% in 2018 compared with 2017 (6,636/3,733). Competing demands over the program continued in 2019, and the first dose coverage dropped (26.9%). For 2020, a municipal law instituted school-based vaccination and the creation of dedicated teams for vaccination, and these strategies are waiting to be tested.

**Conclusion** School-based annual HPV vaccination in children between 9 and 10 years old was feasible and increased vaccination coverage, regardless of gender, although the program was vulnerable to competing events.

## Resumo

**Objetivo** O presente estudo avalia a implantação de um programa de vacinação contra o papilomavírus humano (HPV) em escolas de uma cidade brasileira e o impacto após 2 anos.

**Métodos** Estudo prospectivo para avaliar a implementação do programa, oferecendo a vacina quadrivalente contra o HPV em duas doses anuais, para meninas e meninos de 9 a 10 anos. O programa foi autorizado pelo Programa Nacional de Imunizações na cidade de Indaiatuba, estado de São Paulo, Brasil, e teve início em 2018. A cobertura anual da primeira dose foi comparada ao ano de 2017, e os eventos que influenciaram os resultados foram descritos.

**Resultados** O programa convidou 4.878 crianças por meio das escolas (87,1% da população-alvo) e 7,5% recusou a vacinação. Vários eventos concorrentes exigiram ou competiram pelos profissionais de saúde das equipes de vacinação. A cobertura da primeira dose (9 a 10 anos) foi de 16,1% em 2017 e aumentou para 50,5% em 2018 ( *p* < 0,0001). A primeira dose em todas as idades aumentou 78% em 2018 em comparação com 2017 (6.636/3.733). As demandas concorrentes sobre o programa continuaram em 2019, e a cobertura da primeira dose caiu (26,9%). Para 2020, uma lei municipal instituiu a vacinação nas escolas e a criação de equipes dedicadas à vacinação, e estas estratégias aguardam para ser testadas.

**Conclusão** A vacinação anual contra o HPV em base escolar nas idades de 9 a 10 anos foi viável e aumentou a cobertura vacinal, independentemente do gênero, embora o programa fosse vulnerável a eventos concorrentes.

## Introduction

*Human papillomavirus* (HPV) infection is a necessary condition for the development of cervical cancer, and HPV vaccination prevents up to 90% of this cancer. 1 Screening and vaccination combined are the main strategies towards the eradication of cervical cancer. 2 3 4 5

The World Health Organization (WHO) has recommended HPV vaccination since 2009, starting at the age of between 9 and 10 years old, preferably for girls. 6

The first population program with HPV vaccination started in Australia (2007) and reached a high and sustained coverage reporting a continuous drop in the incidence of cervical cancer precursor lesions. 2 The National Immunization Program (PNI, in the Portuguese acronym) in Brazil offers the quadrivalent HPV vaccine (HPV 6/11/16/18) through the Brazilian Unified Health System (SUS, in the Portuguese acronym) since 2014. The target are girls from 9 to 14 years old, but since 2017 boys from 11 to 14 years old were also included. In the 1 ^st^ year (2014), the strategy was school-based and 5.3 million first doses were applied (coverage of 108%). In the second dose, after a 6-month interval, 3.2 million doses were applied (coverage of 64.8%). 7 In the following year (2015), the strategy changed from school-based to be offered at primary health care facilities, and the PNI reported a significant drop in vaccination coverage: between 50 and 61% in the first dose and between 22 and 38% in the second dose. 8 9

In Indaiatuba, state of São Paulo, Brazil, a school-based HPV vaccine program was planned to increase coverage. The present article describes the rationale, design, implementation, and results of the 1 ^st^ 2 years of the program. Several unexpected competing events prevented the full development of the strategy. The present article is a critical report of the challenges to be overcome in a real-world setting of a middle-income country.

## Methods

This is a prospective study with the objective of evaluating the interventions of a school-based HPV vaccination program aiming to increase coverage in preadolescents and adolescents. In Brazil, the PNI offers HPV vaccine in a two-dose schedule (at a 6-month interval) to girls from 9 to 14 years old and to boys from 11 to 14 years old in primary health care facilities. Indaiatuba, in the state of São Paulo, Brazil, is an urban city with a population of 240,000 inhabitants and a high (0.79) human development index. 10 The municipality launched the school-based program in 2018. The program used the quadrivalent HPV vaccine provided by the PNI (Ministry of Health). The premises and objectives of the program were as follows:

1. **Strategy:** School-based. In Indaiatuba, > 80% of children are students in municipal public schools. The robust public health and educational framework enabled designing the strategy.
2. **Targeted population:** The target population were girls and boys between 9 and 10 years old. The estimated population in 2018 was of 5,600 children from 9 to10 years old and of 11,300 children from 11 to 14 years old. 11 The program designers choose to unify the ages of the target girls and boys to facilitate the logistics of the process. The PNI authorized this change, with the condition that a a surveillance research project were conducted in parallel. The research project was approved by the Ethics Committee of the University of Campinas (CAAE 87358318.2.0000.5404).
3. **Vaccination schedule** : Two annual doses were planned. The first dose was at 9 years old and the second dose at 10 years old. The PNI also authorized this change. The program expected a transition period for those who had already received the first dose at 10 years old. The leaflet of the manufacturer indicates a 12-month vaccination interval (0, 6, and 12 months) from 9 to 15 years old, and this recommendation is adopted by some representative American guidelines. 12
4. **Time for vaccination:** The program designers defined March as the preferred month for immunization since, in Brazil, it is the first month of the academic year. No other competing seasonal vaccination campaign happens during this period. There was also the possibility of integrating the events of ‘Lilac March’, the month of action for cervical cancer awareness and prevention.

The study protocol considered was planned for the years 2017, 2018, and 2019. Data were obtained from the PNI information system, an official recording platform of the Brazilian Ministry of Health. 13 The variables were the number of doses administered per month (first or second dose), by age, and by gender. The program's impact was assessed by calculating the annual coverage. The numerator was the number of doses, and the denominator was the official estimated population for the year, age, and gender. The results related to the years 2018 and 2019 were compared with 2017, just before the program started. The statistical analysis was done by the chi-squared test, with 5% of significance. The implementation steps and results of the program were correlated with competing external events.

## Results

### Preparatory Phase (Year 1, February to August 2018)

The program planners supported by the research team presented the design of the program to the health and education authorities. Seminars about HPV infection, cervical cancer prevention, and immunization safety were presented to stakeholders, such as teachers and other education personnel. According to the Brazilian legislation for the vaccination of children, vaccines on the PNI list do not need authorization from parents or guardians. If the choice is to not vaccinate, a Term of Refusal must be signed. Of the target population, 4,878 children were invited (87.1%), and the Term of Refusal was presented by parents or guardians of 366 children (7.5%).

The vaccination that was scheduled to start earlier, due to some external events, was postponed to start only in September 2018:

1) The outbreak of yellow fever. It happened in March, displacing immunization team workers to the urgent vaccination against it. Considering the seasonal vaccination against influenza in April and May, the beginning of HPV vaccination was planned for June, just before the winter school break.
2) National truckers strike. In May, a national truck driver strike began, with significant logistical repercussions for health supplies. The beginning of the vaccination was then rescheduled for August, after the school break.
3) Measles recrudescence in Brazil. In August, unexpected measles outbreaks were reported in Brazil, triggered by the Latin America immigration crisis, and the PNI launched a national vaccination campaign, replacing the immunization teams.
4) Finally, the HPV vaccination program was started in September, although there was an expectation of competition for human resources due to the 'Pink October Campaign' of breast cancer awareness.

### Vaccination Phase (Year 1, September to November 2018)

**Fig. 1.**
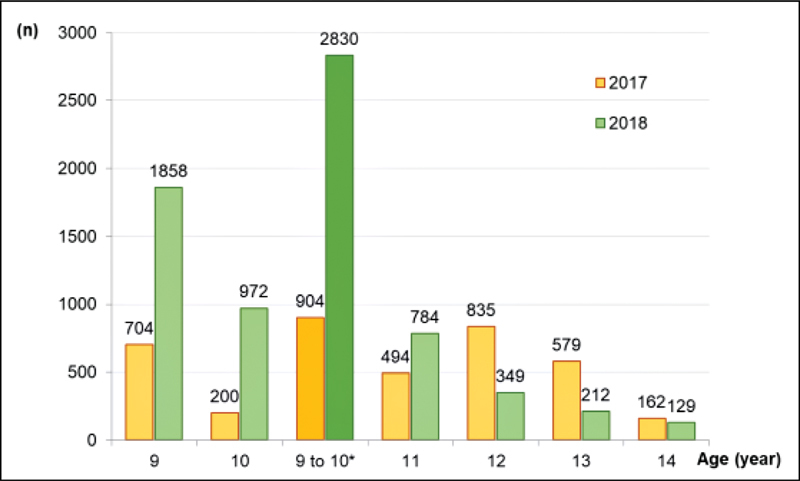
Considering the target ages of the PNI (9 to 14 years old), the first dose of HPV vaccine was given to 4,304 children in 2018 and to 2,974 children in 2017 (Ratio 2018-2017 = 1.45). The difference was due to the higher vaccination rate in the age range between 9 and 10 years old (*Ratio 2018-2017 = 3.1).

**Fig. 2.**
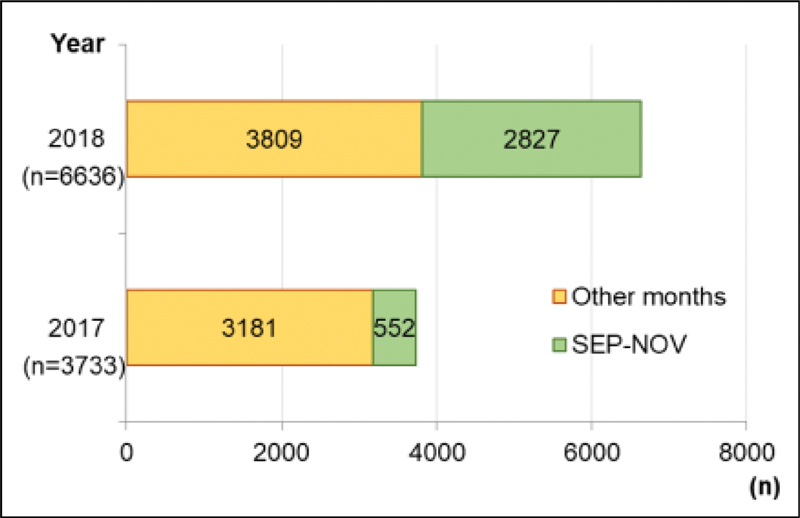
Distribution of the first dose of HPV vaccine applied according to the months of vaccination (school-based vaccination took place from September to November 2018; green bars). Ratio 2018/2017: September-November = 5.12; Other months = 1.20.

Vaccination against HPV in schools was concentrated between September to November. The first dose was given to 2,830 children from 9 to 10 years old, 3 times more than in 2017 ( *n* = 904) for the same age range. The coverage of the first dose (children from 9 to 10 years old) in 2017 and 2018 was, respectively, 16.1 and 50.5% ( *p* < 0.0001). At older ages (12 to 14 years old), the number of first doses given was lower in 2018 than in 2017 ( Figure 1 ). The first dose vaccination in all ages increased 78% in 2018 compared with 2017 (6,636/3,733). It was more concentrated between September and November (ratio = 5.12) when compared with the other months (ratio = 1.20) ( Figure 2 ).

There were no significant questions raised from parents, guardians, or the society. There were no adverse events consistently reported in the period and no serious adverse events were reported.

### Vaccination in 2019 (Year 2)

**Fig. 3.**
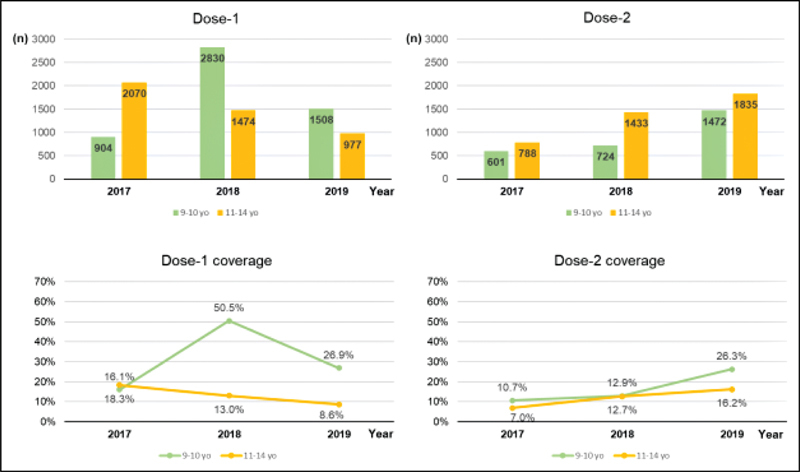
Distribution of Dose1 and Dose 2 of the HPV vaccine administered and coverage by year and age group. School-based vaccination from 2018 at the age range between 9 and 10 years old (yo).

The vaccination against yellow fever, influenza, and measles displaced the immunization health teams throughout the 1 ^st^ semester of 2019. In September, the vaccination campaign in schools resumed, with an evident reduction of interest by all involved. The results were poor, with a significant drop in the number of first doses given to children from 9 to 10 years old ( *n* = 1508; 26.9% coverage) ( Figure 3 ).

### Reassessment of the Vaccination Program

Following the poor results obtained in 2019, a Municipal Law was approved on November 20 ^th^ , instituting the 'Week of HPV vaccination' during the "Lilac March" (Law no. 7255/2019). 14 The objective was to protect the initiative of the HPV vaccination in schools from unexpected events or even unscheduled vaccinations against other diseases.

### Vaccination in 2020 (Year 3)

Considering the obligation by law for City managers to provide vaccination teams dedicated to immunization against HPV, the work in schools was planned for March 2020. In February 2020, there was a recommendation from the PNI to move the influenza vaccination to an earlier date, to March, due to the imminent COVID-19 pandemic. Despite the efforts to keep the schedule, the COVID-19 pandemic arrived, scared the populations, and school activities were suspended in March. In 2020, most immunization programs were interrupted across the country.

### Program for 2021 (Year 4)

There is a commitment by the public managers to achieve the goals proposed by the municipal program, including the vaccination in March 2021, with dedicated teams. However, the COVID-19 pandemic persists and worsens. No presential school activities are planned by now. COVID-19 vaccination has started in January in Brazil, and there is an urgent need to start the seasonal vaccination against influenza before the winter season.

## Discussion

School-based HPV vaccination through a municipal program has proven to be feasible and can increase vaccine coverage. In contrast, the execution of the program presented vulnerabilities due to unexpected events competing for the activities of the immunization team.

In the 1 ^st^ year of the program, even facing several adversities, the age range from 9 to 10 years old achieved 50.5% of coverage with the first dose, a significant increase compared with only 16.1% in 2017. When considering the age range from 12 to 14 years old that was not included in the program, the first doses were kept at low levels. The increased coverage observed in the months of execution of the program compared with the other months and with 2017 indicates the potential success of school-based vaccination.

Countries with high and sustained vaccine coverage, such as Australia, Canada, Sweden, and Malaysia, opted for the school-based strategy. 2 4 5 15 Some of them already demonstrate a progressive effect in the diagnosis of cervical cancer precursor lesions, reporting a decreasing incidence. 2 5 In 2020, a Swedish population assessment demonstrated a substantial reduction in the incidence of cervical cancer in vaccinated women compared to unvaccinated women, especially when vaccinating before 17 years old, with a decrease of 88%. 5

In 2014, the incredible mark of 5.3 million first doses (coverage of 108.7%) was reached in the 1 ^st^ year of the Brazilian program of HPV vaccination by the school-based strategy. The second dose coverage, 6 months later, dropped to 64.8%. 7 This variation in rates is attributed to the media effect raised about safety issues. Supporting global affirmative communication, the World Health Organization (WHO) attests to the safety of the HPV vaccines. Higher levels of antibodies are reported in preadolescents or youngest adolescents, and some protection is expected after the first dose. 16 A 12-month interval for the second dose is recommended, and the risk of failure or of exposure to HPV at these ages is very low, close to 1% in 10-year-old Brazilian girls. 17 18 19 20

The difficulty of the parents in taking adolescents to be vaccinated in Health Care Units during business hours may be the main obstacle and needs to be overcome. This issue is enough to explain the poor results achieved for other ages (from 12 to 14 years old). Therefore, vaccination in schools seems to be the solution.

The school-based strategy used was successful, although it presented points of weakness, some of which were unforeseen. The following aspects need to be highlighted: the availability of  > 80% of children under the tutelage and surveillance of municipal schools; the unification of the target ages for girls and boys; and the annual vaccination schedule. All these strategies are possible to be reproduced across the country.

The program, however, showed its vulnerability to unforeseen situations, mainly due to the lack of a dedicated team for immunization. The attempt for the approval of the Municipal Law by the end of 2019 needs to be tested. It may help to define the human resources to the school-based strategy, reducing the risk of unexpected events.

The COVID-19 pandemic in the year 2020 resulted in no consistent presential school activity, and the vaccination was not carried out. In 2021, with the evident burden and longevity of the COVID-19 pandemic, it is necessary to emphasize that other healthcare actions should not be suspended or postponed but planned to be carried out concurrently. HPV vaccination is essential for the WHO to achieve its goal of accelerating the eradication of cervical cancer through the vaccination of 90% of girls up to 15 years old by 2030. 21

## Conclusion

The school-based HPV vaccination in an organized program significantly increased vaccination coverage in the age range from 9 to 10 years old, regardless of gender, but showing vulnerability to unpredictable or competing events. The creation of dedicated teams prioritizing the execution of the vaccination program is crucial to achieving high coverage. rates.
